# THE ROLE OF COGNITIVE RESERVE IN NEUROINFLAMMATION IN A PANAMANIAN SAMPLE

**DOI:** 10.1002/alz70857_105802

**Published:** 2025-12-25

**Authors:** Sofía A Rodriguez‐Araña, Diana Carolina Oviedo, Adam E. Tratner, Alcibiades E Villarreal, Giselle Rangel, Gabrielle B Britton

**Affiliations:** ^1^ Escuela de Psicología, Universidad Santa María la Antigua (USMA), Panamá, Panamá, Panama; ^2^ Instituto de Investigaciones Científicas y Servicios de Alta Tecnología (INDICASAT AIP), Centro de Neurociencias, Panama, Panama, Panama; ^3^ Sistema Nacional de Investigación (SNI), Panamá, Panamá, Panama; ^4^ Florida State University, Panama, Panama, Panama

## Abstract

**Background:**

Neurodegeneration is linked to cerebrovascular diseases (CVDs) that can impair daily functioning and accelerate cognitive decline in older adults. Blood‐based biomarkers reflect neuroinflammation and cerebrovascular pathology. While many individuals with CVDs experience lasting disabilities, cognitive reserve (CR)—strengthened by lifelong experiences such as education and occupational complexity—can mitigate cognitive decline. However, access to these experiences is more limited in developing countries. The present study aims to explore the relationship between CR, blood‐based biomarkers interleukin‐18 (IL‐18), vascular cell adhesion molecule‐1 (sVCAM1), soluble intercellular adhesion molecule‐1 (sICAM1), and pancreatic polypeptide, and cognition.

**Method:**

The study includes cross sectional analyses from two samples of the Panama Aging Research Initiative‐Health Disparities study (PARI‐HD), consisting of dementia‐free, older adults aged ≥ 65 years (*N* = 429). The first sample (*N* = 181) was recruited during 2012‐2015 from Panama's Social Security Fund geriatric outpatient clinic (M_age_ = 77.4 years, SD = 7.6). The second sample (*N* = 248) was recruited from the community during 2016‐2020 (M_age_ = 71.9 years, SD = 5.6). Global cognition was measured using the Mini Mental State Examination (MMSE). A composite score combining years of education, occupational complexity, and the Instrumental Activities of Daily Living (IADL) score was used to calculate CR.

**Results:**

Multivariate analyses indicated significant sex differences and differences across cohorts. Men were older, had more complex occupations, and had higher concentrations of IL18 and PPP than women. Cohort 1 was significantly older, less educated, and less independent, and had higher concentrations of biomarkers IL18, PPP, sVCAM1, sICAM1 than Cohort 2. Mediation analyses revealed that CR mediates the relationship between sVCAM1 and sICAM1 on global cognition, after controlling for age, sex, and cohort.

**Conclusion:**

These findings demonstrate an important link between neuroinflammatory biomarkers, CR, and cognition among older adults. Public policy targeting enhancing CR may decrease disability associated with neuroinflammation in developing countries like Panama.

